# Body transfer illusions in the schizophrenia spectrum: a systematic review

**DOI:** 10.1038/s41537-022-00314-z

**Published:** 2022-11-23

**Authors:** Kira Baum, Julia Hackmann, Julian Pakos, Kyra Kannen, Annika Wiebe, Benjamin Selaskowski, Max C. Pensel, Ulrich Ettinger, Alexandra Philipsen, Niclas Braun

**Affiliations:** 1 Department of Psychiatry and Psychotherapy, University of Bonn, Bonn, Germany; 2 Department of Psychology, University of Bonn, Bonn, Germany

**Keywords:** Human behaviour, Psychosis, Schizophrenia

## Abstract

Schizophrenia has been viewed as a disorder of the self. Accordingly, the question arises if and how senses of ownership and agency are impaired in schizophrenia. To address this question, several body transfer illusions (BTIs) have been investigated in schizophrenia patients and other schizophrenia spectrum (SCZ-S) populations. The objective of the study was to systematically review the current evidence from BTIs in the SCZ-S. A systematic literature search in PubMed and CENTRAL (search date: February 12, 2022) was conducted on BTI studies carried out in SCZ-S populations. Studies were included if they were published in English after peer review, reported original research data, related to the SCZ-S, and used a BTI as its study method. Conference papers, study protocols, and reviews were excluded. For each included BTI study, various study characteristics and outcomes were retrieved, and a risk-of-bias score was calculated based on six study quality criteria. *K* = 40 studies were identified, of which *k* = 20 studies met the eligibility criteria. For BTI paradigms using visuotactile stimulation, most studies found elevated sense of ownership ratings in SCZ-S populations compared to healthy controls (HC). Implicit illusion measures (e.g., proprioceptive drift), in turn, did not generally indicate elevated embodiment levels in SCZ-S populations. Likewise, no consistent group differences emerged between SCZ-S populations and HC with respect to BTI paradigms using visuomotor stimulation. Furthermore, BTI vividness was found to correlate significantly with core symptoms of schizophrenia and various subclinical characteristics related to the SCZ-S. In line with the self-disturbance hypothesis, SCZ-S populations appear to be affected by aberrations in bodily self-awareness. Review registration: PROSPERO (identifier: CRD42022287960).

## Theoretical background

Although we usually do not actively reflect on ourselves in everyday life, instead taking it pre-reflexively for granted, this tacit self-awareness can be severely disturbed under various clinical conditions^1^. Schizophrenia (SCZ) is a clinical disorder in which one’s own self-awareness may be particularly disturbed^2^. While modern classification systems do not list self-disturbances as a key symptom, presumably due to their atheoretic approach, SCZ has historically been regarded as a disorder of the self. Emil Kraepelin, for instance, considered a disunity of consciousness (an “orchestra without conductor”) to be a central feature of SCZ^3,4^, and Kurt Schneider regarded SCZ as “a loss of the very contours of the self”^5^. Consequently, he added several “ego-disorders” (German: “Ich-Störungen”) to his “first-rank symptoms” of SCZ, that are still in use today. Moreover, in line with these traditional views, more recent SCZ theories like the Ipseity-Disturbance Model (IDM)^2^, have also stressed the importance of self-disturbances in SCZ. Likewise, recent meta-analyses by Burgin et al.^6^ and Raballo et al.^7^ have confirmed a high prevalence of self-disturbances in SCZ patients. Specifically, Burgin et al.^6^ reported a 2.5–12 times higher prevalence of self-disturbances in SCZ and associated conditions as compared to both healthy populations and populations with other mental illnesses. Similarly, Raballo et al.^7^ stated that self-disturbances selectively aggregate in SCZ and associated conditions, have validity as a phenotypic marker of vulnerability to varying degrees of severity of SCZ and, importantly, can be distinguished from a broader proneness to psychosis.

If SCZ indeed involves a disorder of the self, the question arises if and how bodily self-awareness is also affected. In particular, it is not clear to which extent the sense of ownership (SoO) and sense of agency (SoA) are impaired in SCZ, given their ubiquitous involvement in bodily self-awareness^8,9^. Whereas SoO describes an experience of “mineness” toward one’s own feelings, sensations, thoughts, and body parts (e.g., “This hand feels like part of my body”), SoA refers to the authorship experience of initiating and controlling an action (e.g., “It is me who is conducting this button press”)^9^. As such, SoO and SoA are considered subcomponents of embodiment^10^ and several empirical studies suggested that alterations in SoO and SoA are specifically associated with SCZ symptoms^11–13^.

Body transfer illusion (BTI) paradigms are a promising approach to investigate bodily self-awareness. In brief, BTIs can be defined as perceptual illusions that induce SoO and sometimes also SoA over artificial or virtual limbs^1,9,14^. That is, participants undergoing a BTI perceive that a presented artificial or virtual limb belongs to their own biological body (SoO) and that they can control its movements and actions (SoA). By now, various kinds of BTIs have been developed and tested (for an overview, see^9^). The original and most common BTI is the rubber hand illusion (RHI)^15^: by simultaneously stroking both an artificial hand placed in an anatomically plausible position in front of a subject and the subject’s (hidden) real biological hand, an illusory SoO toward the artificial hand can often be evoked^15^. In addition, as an implicit sign of artificial hand embodiment, a so-called proprioceptive drift can frequently be observed, which reflects a shift of the estimated position of the participant’s real hand toward the artificial hand^16^. Interestingly, however, the RHI decays in healthy populations when the artificial hand is positioned in an anatomically incongruent position (e.g., rotated by 180°)^17^, or when the tactile and visual stroking is applied asynchronously^15^. In addition to the RHI, further BTIs have been developed over time, such as the projected hand illusion (PHI)^18^ (i.e., a digital version of the original RHI setup), the mirror box (MB)^19^ (i.e., the induction of SoO/SoA toward a limb’s mirror reflection), and the full-body illusion (FBI)^20^ (i.e., the induction of illusory embodiment toward a whole virtual body). Likewise, besides the original visuotactile induction method (i.e., the participants see the rubber hand being stroked and feel their own hand being stroked), several further BTI induction methods have been explored, including the visuomotor induction method (i.e., participants perform hand movements and simultaneously see a rubber hand imitating these movements). The advantage of this latter induction method is that, in addition to studying SoO and artificial hand embodiment, SoA can also be systematically investigated. For example, the moving RHI paradigm of Kalckert and Ehrsson^21^ examines how the cause of movements of the artificial hand affects SoO and SoA (i.e., whether movements were generated internally or by the experimenter).

Based on the results revealed by these BTI investigations, various neurocognitive theories have been put forward over the years on how and why BTIs emerge (for reviews, see^9,14,22^). In short, and following Tsakiris’ taxonomy^22^, these theories can be arranged on a continuum between bottom–up and top–down approaches. Whereas bottom–up accounts suggest that successful BTI induction mainly depends on multisensory integration (e.g., matching visual and tactile sensory information in case of the classical RHI) and only marginally on internal body maps, top–down accounts assume stronger involvement of internal body maps (e.g., that an internal model about the hand’s shape and color exists that determines which perceptual objects can be experienced as part of the own body).

Originally, BTIs were primarily tested in healthy populations, but more recently they have also been studied in patients with SCZ as well as in populations with related clinical or subclinical conditions. With the exception of a mini meta-study^20^ that was limited to the question of whether SCZ patients have a higher susceptibility to bodily illusions than healthy controls (HC), these BTI studies have not been systematically summarized and synthesized yet. Therefore, the aim of this preregistered systematic review is to summarize, link, and evaluate previous findings on BTIs in individuals with SCZ and related (sub)clinical conditions, as well as to give an overview of open research questions and potential clinical applications. To comprehensively review the entire body of BTI literature available in the schizophrenia spectrum (SCZ-S), the current work also includes studies that examined individuals with schizoaffective disorder, high psychosis- or delusion-proneness, or high levels of schizotypy. Of course, not all of these states are pathological, and the question rightfully arises to what extent firm conclusions about SCZ can be drawn for instance from subclinical SCZ-S states, such as high schizotypy (for a comprehensive discussion, see^23,24^). In the present review, the umbrella term SCZ-S is used whenever statements refer not exclusively to SCZ but also to schizoaffective disorder or any subclinical condition (high levels of schizotypy, high psychosis-proneness, high delusion-proneness). Wherever applicable, however, the specific population under investigation will be mentioned.

## Methods

### Protocol and registration

This systematic review was conducted in accordance with the Preferred Reporting Items for Systematic Reviews and Meta-Analyses^25^ and preregistered in PROSPERO (identifier: CRD42022287960).

### Information sources and search strategy

A systematic literature search on titles, abstracts, and keywords was performed in PubMed and CENTRAL (search date: February 12, 2022). A conjunctive search query was made, whose first operand contained a disjunctive set of keywords (schizo*, psychosis, psychotic, psychosis-proneness) relating to clinical and subclinical SCZ-S phenotypes. Appropriate truncations were hereby used to search for all variations of a word stem. The second operand searched for BTI paradigms and contained a disjunctive set of relevant keywords (rubber hand illusion*, virtual hand illusion*, projected hand illusion*, full body illusion*, body transfer illusion*).

### Eligibility criteria

To be eligible, a study had to be published in English, to be peer-reviewed, to report original research data, to be SCZ-S-related, and to have used a BTI as its study method. Conference papers, study protocols, and reviews were excluded. No inclusion restrictions were made with respect to sample age, BTI paradigm implemented, study outcomes, or control conditions.

### Study characterization and evaluation

For each included study, various characteristics were retrieved. Moreover, to assess the study quality, a risk of bias (RoB) score (in %) was calculated, based on the following Yes/No criteria:

1. Ordering/assignment-control: Was it ensured that the BTI conditions under comparison were (pseudo-)randomized or counterbalanced?
2. Control group: Was there a HC group to which the SCZ-S sample was compared?
3. Control condition: Was there a control condition (e.g., asynchronous stroking, passive movement), to which the actual BTI condition (e.g., synchronous stroking, active movement) was compared, in order to detect general suggestibility effects?
4. Control items: Did the BTI questionnaire include a statistical comparison to control questions to detect general suggestibility effects and response biases?
5. Registration: Was the study enrolled in an official study register?
6. Blinding: Were the study staff blinded with regard to the different intervention groups?

A RoB of 100% (no criteria fulfilled) was defined to indicate a low study quality and a RoB of 0% (all criteria fulfilled) a high study quality.

### Data inspection

Study eligibility assessments and RoB evaluations were performed by two independent reviewers (J.H., K.B.), and disagreements were resolved by discussion or by consulting a third reviewer (N.B.).

## Results

**Fig. 1:**
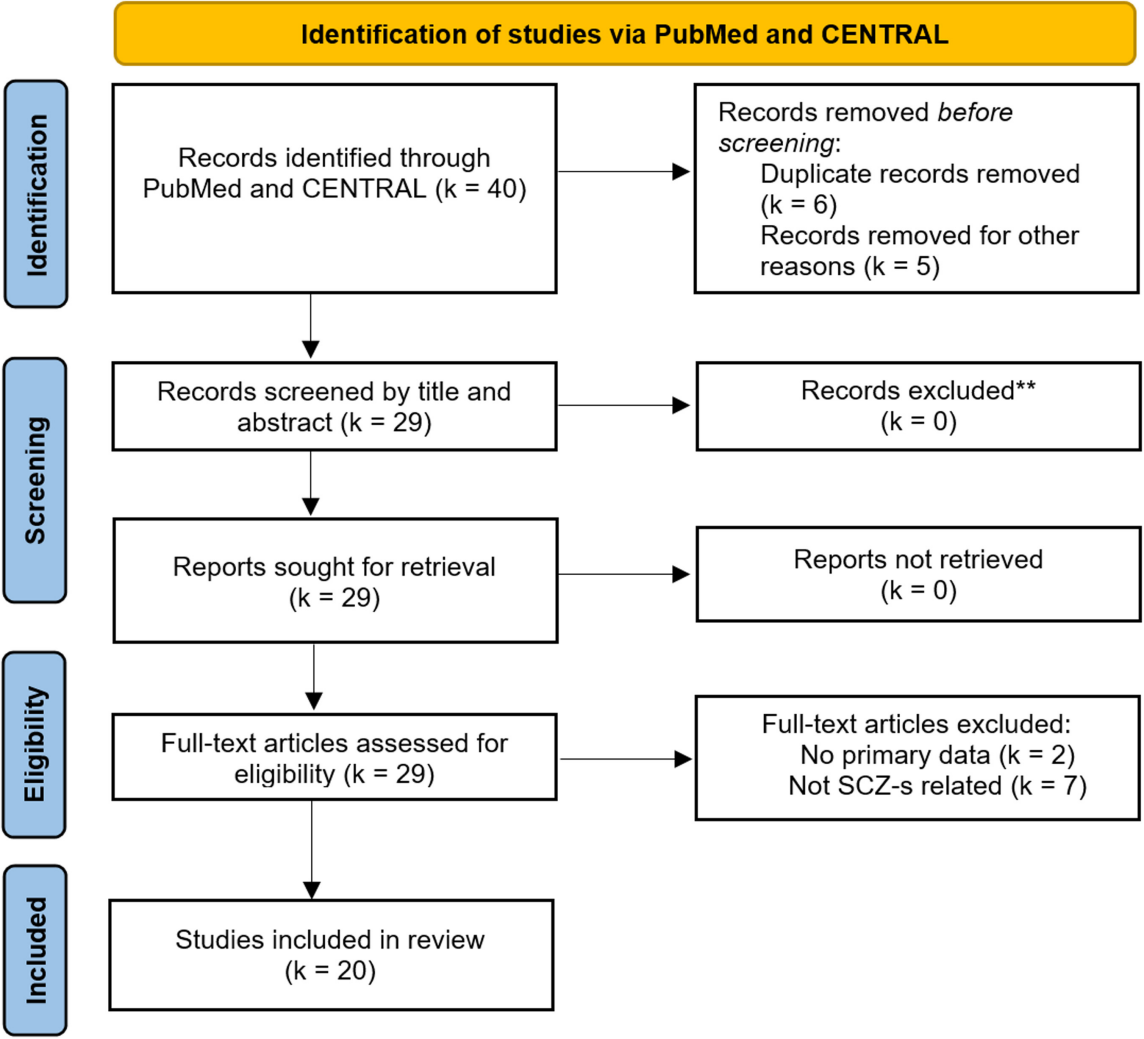
PRISMA flow diagram illustrating the study selection process.

Based on our literature search, a total of *k* = 40 studies were identified, out of which *k* = 20 studies were deemed eligible and included in the review (see PRISMA flowchart in Fig. 1).

The excluded *k* = 20 studies did not report primary data (*k* = 2), were not related to the SCZ-S (*k* = 7), duplicates (*k* = 6), or were excluded for other reasons (*k* = 5). The main study characteristics and results of included studies are summarized in Table 1. There was high variability with respect to study quality, examined SCZ-S population, operationalized BTI paradigm, and measured BTI parameters. Pertaining study quality, the estimated overall RoB was quite high (*M* = 60%) but varied considerably (*SD* = 18.26%) between studies and paradigms (for further details, see SM1). Regarding SCZ-S populations, studies were performed in patients with SCZ (*k* = 11) and in mixed groups of patients with SCZ or schizoaffective disorder (*k* = 4), varying degrees of schizotypy (*k* = 2), psychosis-proneness (*k* = 1), psychotic-like experiences (*k* = 1), or delusion-proneness (*k* = 1). Concerning the BTI paradigms that were implemented, *k* = 11 studies relied on the classical RHI, *k* = 3 on a PHI version, *k* = 1 on the MB paradigm, *k* = 1 on the FBI, *k* = 2 on the moving RHI, and *k* = 2 on an otherwise modified RHI. Regarding BTI parameters, most studies collected phenomenological data based on standardized BTI questionnaires (*k* = 19). Implicit embodiment measures such as the proprioceptive drift (*k* = 8), somatosensory evoked potentials via electroencephalography (EEG) (*k* = 1), skin temperature (*k* = 1), the self-localization drift (*k* = 1), or the forearm bisection task (*k* = 1) were, in turn, used less often. Except for two studies in which skin temperature and EEG were obtained, no other physiological embodiment measures, such as the electrodermal fear response during artificial limb threatening^26–28^, were collected.

**Table 1:** Summary of identified BTI studies conducted on the schizophrenia spectrum.

Study	Study population				Study characteristics				Results
	Sample	*N* (male)	Age	Diagnostic instrument	Paradigm	Condition	Risk of bias^a^ (in %)	Illusion measure	Outcome
Peled et al. (2000)^35^	Adults with schizophrenia and healthy adults	SCZ = 26 (20) HC = 23 (10)	*M* = 36; *SD* = 7.2 *M* = 40; *SD* = 10.7	SCID-IV, SAPS, SANS	Classical RHI	A: Synch. visuotactile stimulation	83	Self-report questionnaire: SoO	Greater illusion strength and faster illusion onset in SCZ than HC Positive correlation between two SoO items and hallucinations
Peled et al. (2003)^36^	Adults with schizophrenia and healthy adults	SCZ = 19 (16) HC = 19 (17)	*M* = 32; *SD* = 10 *M* = 25; *SD* = 8.7	DSM-IV diagnosis, PANSS	Classical RHI	A: Synch. isuotactile stimulation	83	Self-report questionnaire: SoO	Greater illusion strength in SCZ than HC
								EEG	Healthy adults showed significant electrophysiological differences in various somatosensory evoked potential (SEP) components between SEPs recorded before RHI experience and SEPs recording during RHI experience, adults with schizophrenia did not show such alterations
Asai et al. (2011)^49^	Healthy adults	HC = 72 (36)	*M* = 19.7; (18–24)	SPQB	Classical RHI	A:Synch.visuotactile stimulation	83	RHI sensitivity (averaged proprioceptive drift and SoO questionnaire)	Significant correlation between RHI sensitivity and positive schizotypy Positive moderate correlation between SoO questionnaire and proprioceptive drift
Thakkar et al. (2011)^31^	Adults with schizophrenia and healthy adults	SCZ = 24 (15) HC = 21 (11)	*M* = 41.7; *SD* = 8.3 *M* = 40.1; *SD* = 9.1	SCID-IV, BPRS, SAPS, SANS, SPQ	Classical RHI	A: Synch. visuotactile stimulation B: Asynch. visuotactile stimulation	50	Self-report questionnaire: Embodiment	Main effect of group (higher scores in SCZ) Main effect of synchronicity (higher scores after synch. condition) Correlation between RHI score with positive and negative schizotypy in HC (for synch. and asynch. condition) Correlation between SoO score and hallucination subscale in SCZ (synch. condition only)
								Proprioceptive drift	Main effect of group (greater proprioceptive drift in SCZ) Main effect of synchronicity (greater proprioceptive drift in synch. condition) Group × synchronicity interaction (greater proprioceptive drift in SCZ after synch. but not asychn. condition)
								Skin temperature	No difference between groups
Germine et al. (2013)^46^	Healthy adults	HC = 55 (20)	*M* = 28; *SD* = 11	MINI clinical interview, subscales for positive- and negative psychosis-proneness	Classical RHI	A: Synch. visuotactile . stimulation B: Asynch. visuotactile stimulation	83	Self-report questionnaire: SoO and SoA	Main effect of synchronicity (higher SoO and SoA scores after synch. condition) Significant correlation between positive psychosis-proneness and SoO score in synch. condition
								Proprioceptive Drift	Main effect of synchronicity (greater proprioceptive drift in synch. condition)
Ferri et al. (2014)^40^	Adults with schizophrenia and healthy adults	SCZ = 21 (21) HC = 17 (17)	*M* = 41.1; *SD* = 11.4 *M* = 46.6; *SD* = 13.9	SCID-I (for SCZ) BPRS, SAPS, SANS, SCID-II (for HC)	RHI variant	A: Rubber Hand-Congruent (RH-C) B: Rubber Hand Incongruent C: Wood Congruent D: Wood Incongruent	50^b^	Self-report questionnaire: SoO	Higher SoO score in HC for RH-C condition Highest mean rating in RH-C for SCZ and HC compared to all other conditions Correlation between SoO score and SANS anhedonia scale in SCZ for RH-C condition
Graham et al. (2014)^18^	Adults with schizophrenia or schizoaffective disorder Sub-division: never, past, or current passivity symptoms and healthy adults	SCZ = 53 (36) HC = 48 (24)	Never: *M* = 42.5; *SD* = 1.6 Past: *M* = 43.6; *SD* = 2.8 Current: *M* = 44.0; *SD* = 2.1 *M* = 46.2; *SD* = 1.68	SAPS, SANS, PSI, SCAN	Projected Hand Illusion	A: Synch. visuotactile stimulation B: Asynch. visuotactile stimulation	50^b^	Self-report questionnaire: SoO, SoA, disembodiment of own hand, loss of agency over own hand	No overall main effect of group for SoO and SoA ratings Higher scores of disembodiment and loss of agency in SCZ Group × synchronicity interaction (Current group showed equal SoO scores for synch. and asynch. condition) Main effect of synchronicity for SoO and SoA ratings
Graham et al. (2014)^47^	Healthy adults	HC = 48 (24)	*M* = 46.2; *SD* = 11.6	Psychotic-like experience (PLE) questionnaire	Projected Hand Illusion	A: Synch. visuotactile stimulation B: Asynch. visuotactile stimulation	67	Self-report questionnaire: SoO, SoA, disembodiment of own hand, deafference	Main effect of PLE (higher PLE score associated with higher overall illusion rating (SoO, SoA, disembodiment, and deafference)
								Proprioceptive drift	No effect of PLE
Kaplan et al. (2014)^32^	Adults with schizophrenia or schizoaffective disorder, adults with body dismorphic disorder (BDD) and healthy adults	SCZ = 17 (4) BDD = 17 (4) HC = 17 (4)	*M* = 37.1; *SD* = 10.3 *M* = 39.5; *SD* = 10.0 *M* = 35.4; *SD* = 9.7	MINI, SAPS, SANS, Perceptual Aberration Scale, Somatic Symptoms Scale, Social Fear Scale	Classical RHI	A: Synch. visuotactile stimulation B: Asynch. visuotactile stimulation	33	Self-report questionnaire: Embodiment Proprioceptive drift	Higher mean ratings in SCZ in three embodiment items Main effect of synchronicity (higher embodiment scores in synch. condition)
									Group × synchronicity interaction
Kállai et al. (2015)^13^	Healthy adults	HC = 48 (20)	*M* = 20.9; *SD* = 2.01	SCL-90-R	Classical RHI	A: Synch. visuotactile stimulation B: Asynch. visuotactile stimulation	33	Self-report questionnaire: SoO Proprioceptive drift	Significant main effect of synchronicity (higher scores in synch. condition) Large positive correlation between SoO and psychoticism (vulnerability to schizotypy) in synch. condition
									Main effect of synchronicity (higher scores in synch. condition) No significant correlations with SCL-90-R scales
Lev-Ari et al. (2015)^33^	Adults with schizophrenia and healthy adults	SCZ = 30 (24) HC = 30 (15)	*M* = 37.4; *SD* = 11.2 *M* = 30.9; *SD* = 12.6	SCID (DSM-IV), BPRS	Classical RHI	A: Synch. visuotactile stimulation (on 3 trial days)	83	Self-report questionnaire: SoO	Group × trial interaction (no group difference in trial 1; higher SoO score in HC in trial 2 and 3)
Louzolo et al. (2015)^38^	Healthy adults	HC = 71 (30)	*M* = 24.3 (18–42)	PDI	Moving RHI	A: Active synch. visuomotor stimulation B: Active asynch. visuomotor stimulation C: Passive synch. visuomotor stimulation D: Passive asynch. visuomotor stimulation	50	Self-report questionnaire: SoO and SoA (averaged as self-recognition score)	Moderate positive correlation between self-recognition score and delusion-proneness in passive conditions No correlation between self-recognition score and delusion-proneness in active conditions
Mirucka (2016)^34^	Adults with schizophrenia and healthy adults	SCZ = 31 (17) HC = 33 (10)	*M* = 28.4; *SD* = 5.9 *M* = 24.7; *SD* = 2.6	ICD-10 diagnosis	Classical RHI	A: Synch. visuotactile stimulation	83	Self-report questionnaire: SoO	Main effect of group (higher SoO scores in SCZ)
Graham-Schmidt et al. (2018)^39^	Adults with schizophrenia or schizoaffective disorder Sub-division: never, past, or current passivity symptoms and healthy adults	SCZ = 51 (35) HC = 47(24)	Never: *M* = 42.5; *SD* = 1.6 Past: *M* = 42.6; *SD* = 3.2 Current: *M* = 44.0; *SD* = 2.1 *M* = 45.9; *SD* = 1.7	SANS, SAPS, Passivity Symptoms Interview, SCAN	Projected Hand Illusion	A: Active synch. visuomotor stimulation B: Active asynch. visuomotor stimulation C: Passive synch. visuomotor stimulation D: Passive asynch. visuomotor stimulation	67^b^	Self-report questionnaire: SoA	Condition (active/passive) × delay (synch./asynch.) interaction (reduced SoA after asynch. in active, but not passive condition in all participants) Reduced SoA in active asynch. condition in HC and Past group (but not in Never and Current group)
Shaqiri et al. (2018)^20^	Adults with schizophrenia	SCZ = 59 (44) HC = 30 (16)	*M* = 36.5; *SD* = 9.5 *M* = 36.9; SD = 8.0	Diagnostic interview based on SCID (DSM-IV), SANS, SAPS	Full-Body Illusion	A: Synch. visuotactile stimulation B: Asynch. visuotactile stimulation	33	Self-report questionnaire: SoO	Main effect of group (higher SoO scores in SCZ) Main effect of question type (higher scores in experimental compared to control questions) Main effect of synchronicity (higher scores in synch. condition)
								Global self-localization drift	No effect of group or synchronicity
Prikken et al. (2019)^29^	Cohort 1:								
	Adults with schizophrenia and healthy adults	SCZ = 54 (46) HC = 56 (52)	*M* = 34.08; *SD* = 7.98 *M* = 33.84; *SD* = 8.04	SELF, PANSS	Classical RHI	A: Synch. visuotactile stimulation B: Asynch. visuotactile stimulation	50	Self-report questionnaire: SoO	Main effect of synchronicity (higher SoO scores in synch. condition) Group × synchronicity interaction (synchronicity effect smaller in SCZ) Moderate correlation between PANSS delusions and SoO score in synch. condition
								Proprioceptive drift	Main effect of synchronicity (greater proprioceptive drift in synch. condition)
	Cohort 2:								
	Children/adolescents with increased familiar risk of schizophrenia, children/adolescents with increased familiar risk of mood disorder and healthy children/adolescents	SCZ-risk = 24 (6) MD- risk = 33 (15) HC = 18 (13)	*M* = 16.9; *SD* = 2.4 *M* = 17.7; *SD* = 2.5 *M* = 16.06; *SD* = 2.6	K-SADS-PL	Classical RHI	A: Synch. visuotactile stimulation B: Asynch. visuotactile stimulation	50	Self-report questionnaire: SoO	Main effect of synchronicity (higher SoO scores in synch. condition)
								Proprioceptive drift	Main effect of synchronicity (greater proprioceptive drift in synch. condition)
Costantini et al. (2020)^48^	Adults with schizophrenia and healthy adults	SCZ = 22 (22) HC = 22 (22)	*M* = 42, *SD* = 10 *M* = 39, *SD* = 13 not subject to RHI	SCID (DSM-V), SAPS, SANS, BSABS, SPI-A	Classical RHI	A: Congruent synch. visuotactile stimulation B: Congruent asynch. visuotactile stimulation C: Incongruent synch. visuotactile stimulation	67	Proprioceptive drift	Main effect of induction method (greater proprioceptive drift after congruent synch. visuotactile stimulation than incongruent synch. visuotactile stimulation and congruent asynch. visuotactile stimulation)
Rossetti et al. (2020)^19^	Adults with schizophrenia and healthy adults	SCZ = 29 (18) HC = 36 (6)	*M* = 41.3; *SD* = 14.1 *M* = 25.8 *SD* = 7.9	SCID for DSM-IV-TR, SANS, SAPS	Mirror Box	A: In-Phase (synch.) visuomotor stimulation B: In-Antiphase (asynch.) visuomotor stimulation C: Random visuomotor stimulation	50	Self-report questionnaire: Embodiment with subscales for SoO, SoA, Location	Main effect of synchronicity for overall embodiment, SoO, and SoA (higher scores in synch. condition) Group × synchronicity interaction for SoA Moderate positive correlation between hallucination severity with SoO, SoA, and Location in random condition
								Forearm Bisection Task	HC: Distal shift (illusion index) in In-Phase condition and proximal shift (non-illusion index) in the control conditions (In-Antiphase and Random) SCZ: Moderate proximal shift in all conditions
Zopf et al. (2021)^30^	Adults with schizophrenia or schizoaffective disorder and healthy adults	SCZ = 23 (15) HC = 21 (12)	*M* = 49.0; *SD* = 8.4 *M* = 50.1; *SD* = 11.1	DIP, SAPS, SANS, PAS, SPQB, Screening SCID (DSM-IV) for HC	RHI variant (manual brush stroking+ LED/tactor)	A: Synch. visuotactile stimulation (brush) B: Asynch. visuotactile stimulation (brush) C: Synch. visuotactile stimulation (LED/tactor) D: Slightly asynch. visuotactile stimulation (LED/tactor) E: Asynch. visuotactile stimulation (LED/tactor)	50	Self-report questionnaire: SoO, SoA, and Location (averaged)	Main effect of group (higher scores for SCZ in all conditions) Main effect of synchronicity (highest scores for synch. manual brushing) Moderate association between body-related perceptual symptoms and illusion rating
								Proprioceptive drift	No significant effects
Laurin et al. (2021)^37^	Adults with schizophrenia with first-rank symptoms (FRS+) or without first-rank symptoms (FRS-)	FRS + = 31 (23) FRS- = 25 (11)	*M* = 37.7; *SD* = 10.5 *M* = 43.4; *SD* = 12.1	Semi-structured interview based on DSM-IV, DIGS, SAPS, SANS	Moving RHI	A: Active synch. visuomotor stimulation B: Active asynch. visuomotor stimulation C: Passive synch. visuomotor stimulation	50	Self-report questionnaire: SoO and SoA	Both FRS- and FRS+ report SoO and SoA in active synch. visuomotor stimulation and passive synch. visuomotor stimulation condition FRS- report SoA in active asynch. visuomotor stimulation condition, while FRS+ did not

*Asynch.* asynchronous, *BDD* body dismorphic disorder, *BPRS* Brief Psychiatric Rating Scale, *BSABS* Bonn Scale for the Assessment of Basic Symptoms, *DIGS* Diagnostic Instrument for Genetic Studies, *DIP* Diagnostic Interview for Psychosis, *DSM-IV* Diagnostic and Statistical Manual of Mental Disorders—Fourth Edition, *EEG* electroencephalogram, *FRS+* adults with schizophrenia with first-rank symptoms, *FRS-* adults with schizophrenia without first-rank symptoms, *HC* healthy controls, *ICD-10* International Classification of Diseases 10th Revision, *K-SADS-PL* Schedule for Affective Disorders and Schizophrenia for School-age Children—Present and Lifetime Version, *LED* light emitting diode, *MD* mood disorder, *MINI* MINI International Neuropsychiatric Interview, *PANSS* Positive and Negative Symptom Scale, *PAS* Chapman Perceptual Aberration scale, *PDI* Peters’ Delusion Inventory, *PLE* psychotic-like experiences, *PSI* Passivity Symptoms Interview, *RH-C* Rubber Hand-Congruent, *RHI* rubber hand illusion, *SCZ* schizophrenia, *SANS* Scale for Assessment of Negative Symptoms, *SAPS* Scale for Assessment of Positive Symptoms, *SCAN* Schedule for Clinical Assessment in Neuropsychiatry, *SCID* Structured Clinical Interview for DSM-IV Axis I Disorders, *SCID-I* Structured Clinical Interview for DSM-IV Axis I Disorders, *SCID-II* Structured Clinical Interview for DSM-IV for Axis II Personality Disorders, *SCID-IV* Structured Clinical Interview for DSM-IV, *SCL-90-R* Symptom Checklist-90-R, *SELF* Self-experience Lifetime Frequency Scale, *SEP* sensory evoked potential, *SoA* sense of agency, *SoO* sense of ownership, *SPI-A* Schizophrenia Proneness Instrument for Adults, *SPQ* Schizotypal Personality Questionnaire, *SPQB* Schizotypal Personality Questionnaire Brief, *synch.* synchronous, *tactor* tactile stimulator.

^a^A Risk of Bias (RoB) of 100% indicates a low study quality, an RoB of 0% indicates a high study quality.

^b^Control items were collected but not evaluated to check for response bias. The RoB criterion was nevertheless considered to be fulfilled.

In terms of content, the studies primarily addressed four research questions, with some addressing more than one at a time: (1) Do populations of the SCZ-S display stronger SoO and overall embodiment levels toward BTIs than HC? (2) Do SCZ-S populations show differences in SoA during BTI inductions? (3) How relevant is multimodal synchronicity for BTI induction in SCZ-S individuals? (4) Are there associations between SCZ-S symptoms and BTI parameters? The results of the included studies are presented following these four main questions. Regarding self-report data, studies differed in their inclusion and analysis of questionnaire items. For example, while some studies specifically analyzed SoO toward the artificial limb, others examined more general facets of BTI embodiment. Therefore, in the following, questionnaire items are only considered as SoO ratings if they exclusively addressed a mineness experience toward the artificial limb or some location shift toward the artificial hand(e.g., ^29^). If multiple facets of embodiment (e.g., SoO and SoA items) were in turn collapsed into one general rating, the term embodiment rating will be used (e.g., ^30^).

### Research question 1: Do populations of the SCZ-S display stronger SoO and overall embodiment levels toward BTIs than HC?

In total, *k* = 15 studies addressed the question of whether SCZ-S individuals are more susceptible to BTIs than HC. Out of these, *k* = 10 studies based their investigation on visuotactile BTIs, whereby *k* = 8 used an RHI^29–36^, *k* = 1 an FBI^20^ and *k* = 1 a PHI setup^18^. Another *k* = 4 studies relied on visuomotor BTIs, whereby *k* = 2 applied the moving RHI^37,38^, *k* = 1 the MB paradigm^19^ and *k* = 1 a visuomotor PHI^39^. The remaining study applied a modified RHI in which neither tactile nor motor stimulation was applied^40^.

#### BTI investigations based on visuotactile stimulation

Regarding visuotactile BTIs, a majority of studies found that individuals within the SCZ-S show higher SoO ratings during BTI induction than HC. More specifically, out of the *k* = 11 studies addressing this research question, *k* = 7 studies found evidence for higher SoO ratings^20,34–36^ or embodiment ratings^30–32^ in individuals within the SCZ-S, *k* = 2 studies found the opposing effect (i.e., lower SoO ratings^33,40^ in individuals within the SCZ-S) and *k* = 2 studies did not find any group differences^18,29^.

Of the studies that found higher SoO or embodiment ratings in SCZ-S populations, Thakkar et al.’s RHI study^31^ reported higher embodiment scores for both synchronous and asynchronous stroking, whereas the RHI studies by Mirucka^34^ and Peled et al.^35,36^ solely implemented a synchronous visuotactile RHI condition and reported higher SoO scores. The RHI study by Zopf et al.^30^, in turn, not only conducted classical synchronous and asynchronous stroking conditions, but also (a)synchronous conditions applied with an LED (for visual feedback) and a tactile stimulator (for tactile feedback) attached to the participants’ index fingers. Here, higher embodiment ratings were found across all conditions in the SCZ group. Finally, Shaqiri et al.^20^ implemented an FBI and found higher SoO ratings toward a whole virtual body among patients with SCZ compared to HC. However, the authors did not interpret these results as a specific indication for altered SoO of individuals with SCZ, given that additional control items assumed to be unrelated to body ownership were also rated higher in the patient sample and there was no significant three-way interaction between question type (experimental vs. control questions), synchrony (synchronous vs. asynchronous condition), and group (SCZ vs. HC)^20^.

Among the studies finding opposite effects, Ferri et al.^40^ reported lower SoO ratings in individuals within the SCZ-S. However, the RHI induction method was quite different in that study: Instead of actually undergoing hand strokes, participants only observed the experimenter’s hand approaching the artificial hand under anatomically congruent and anatomically incongruent RHI conditions. While in the congruent condition, which yielded the strongest SoO ratings in both groups, the SCZ group reported lower SoO ratings compared to the HC group, no group differences were found in the incongruent RHI conditions^40^. In the other study, Lev-Ari et al.^33^ implemented the synchronous RHI condition on three trial days to test for a potential illusion learning effect. On the first trial day, both groups reported similar SoO ratings, while in subsequent trials more individuals of the HC group than the SCZ group showed an SoO learning effect (i.e., higher SoO ratings in later trials).

Regarding the aforementioned null findings, Prikken et al.^29^ applied the classical RHI and did not find a group difference in SoO. Graham et al.^18^, in turn, applied the PHI to a mixed population of patients with SCZ or schizoaffective disorder and also did not find a significant SoO difference compared to HC.

In contrast to these self-rating results, hardly any evidence for an increased BTI susceptibility of individuals with SCZ-S has been found in implicit embodiment measures. While *k* = 1 study reported a higher proprioceptive drift in individuals within the SCZ-S^31^, *k* = 4 studies^20,29,30,32^ did not find any proprioceptive drift group differences. A potential explanation for this discrepancy between SoO rating and proprioceptive drift results might be that both measures address different aspects of artificial limb embodiment^41–45^. For instance, Gallagher et al.^43^ hypothesized that subjective questionnaires reflect cognitive processes such as the personal body image, while the proprioceptive drift reflects the integration of multisensory stimuli (e.g., visual and tactile feedback). Following this approach, explicit and implicit illusion measures assess distinct aspects of bodily self-awareness that both are complementary and of individual importance.

#### BTI investigations based on visuomotor stimulation

Regarding visuomotor BTIs, the few conducted studies revealed no clear indication of greater susceptibility toward BTIs in individuals within the SCZ-S compared to HC. While the studies by Louzolo et al.^38^, Graham-Schmidt et al.^39^ and Laurin et al.^37^ did not compare individuals within the SCZ-S and HC, the study by Rossetti et al.^19^ found lower illusion experiences in the implicit measure among individuals within the SCZ-S. More specifically, Rossetti et al.^19^ applied the MB paradigm, in which the participants performed movements with their hidden hand while observing the experimenter’s synchronous (In-Phase), asynchronous (Anti-Phase), or random hand movements in a mirror. As an implicit measure, the forearm bisection task was used during which the participants were instructed to blindly point at the midpoint of their tested forearm. A successful illusion induction was assumed if a distal shift (i.e., estimated midpoint shifted toward the tested hand) was observed. Here, the SCZ group showed a proximal shift in all movement conditions, while the HC group showed a distal shift in the In-Phase condition.

### Research question 2: Do SCZ-S populations show differences in SoA during BTI inductions?

In contrast to the multitude of BTI studies assessing SoO, only *k* = 7 studies also separately surveyed SoA. Of those, *k* = 3 relied on visuotactile stimulation^18,46,47^ and *k* = 4 on visuomotor stimulation^19,37,38,39^.

*K* = 3 studies^18,19,39^ found less sensitivity to visuomotor incongruency (i.e., similar levels of SoA under both synchronous and asynchronous stimulation) in SCZ patients in general and in different SCZ subgroups. Rossetti et al.^19^ used a visuomotor MB paradigm in which the level of multisensory synchrony was systematically varied across a synchronous, an asynchronous, and a random visuomotor feedback condition. While in the HC group SoA turned out to diminish with declining synchrony, it remained constant across conditions in the SCZ group. Due to data transformation to counteract possible response biases, no conclusion about the actual SoA level experienced in the different study conditions was possible. In line with this, Graham et al.^18^ found that individuals with current passivity symptoms reported statistically similar degrees of SoA under both a synchronous and an asynchronous visuotactile PHI condition, while HC report decreased SoA after asynchronous stimulation. Graham-Schmidt et al.^39^ extended these results by applying a PHI but providing visuomotor instead of visuotactile feedback. They found that both patients who currently showed passivity symptoms and those who never experienced passivity symptoms reported similar levels of SoA after synchronous and asynchronous visuomotor induction, while HC and patients with past passivity symptoms showed significantly lower SoA under visuomotor asynchrony.

Laurin et al.^37^, in turn, used a moving RHI paradigm to compare SCZ patients with and without first-rank symptoms. The authors thereby referred to the original definition by Schneider^5^, which specified auditory hallucinations, thought broadcast, insertion, and withdrawal, as well as delusional perceptions as first-rank symptoms. While, on average, SCZ patients with first-rank symptoms reported no SoA over the artificial hand in an asynchronous movement condition, SCZ patients without first-rank symptoms did report SoA in that study.

The remaining three studies explored potential relationships between SoA and delusion-proneness^38^, SoA and psychosis-proneness^46^, and SoA and psychotic-like experiences^47^. Germine et al.^46^ applied the classical visuotactile RHI and found a significant correlation between psychosis-proneness and SoA under both visuotactile synchrony and asynchrony. As the authors noted, however, these correlation results for the asynchronous condition could have also derived from a carry-over effect, given that the asynchronous stimulation always followed the synchronous condition. Using a moving RHI, Louzolo et al.^38^ reported a significant correlation between SoA and delusion-proneness in passive (synchronous and asynchronous) but not in active conditions. Finally, Graham et al.^47^ found no specific effects of psychotic-like experiences on SoA in their applied visuotactile PDI.

In sum, some evidence exists that SCZ patients in general, some specific SCZ subpopulations (e.g., SCZ patients without first-rank symptoms or with present passivity symptoms) as well as some SCZ-S populations (e.g., individuals with psychosis- or delusion-proneness) show “over-inclusive agency”^38^ for asynchronous BTI conditions, where SoA usually diminishes. However, given that conclusions are largely based on null findings, (unplanned) subgroup analyses, and self-report data, these findings should be interpreted with caution. Further BTI studies on SoA with more rigorous experimental designs are therefore necessary.

### Research question 3: How relevant is multimodal synchronicity for BTI induction in SCZ-S individuals?

In addition to investigating whether individuals within the SCZ-S show higher susceptibility toward BTIs, *k* = 10 visuotactile^13,18,20,29–32,46–48^ and *k* = 2 visuomotor BTI studies^19,39^ also examined the role of multimodal synchronicity in the induction of BTIs in individuals within the SCZ-S.

Concerning explicit illusion measures, *k* = 7 studies found higher SoO ratings^13,18–20,29,46,47^, *k* = 3 studies found higher embodiment ratings^30–32^ and *k* = 3 studies found higher SoA ratings under synchronous than asynchronous stroking within individuals of the SCZ-S^18,19,46^. However, in the study by Graham-Schmidt et al.^39^, this SoA effect was observed only under active but not under passive visuomotor stimulation, and *k* = 2 other studies^18,19^ also found merely similar levels of SoA (in some subgroups) in SCZ individuals after synchronous and asynchronous induction. Also, *k* = 4 studies found that patients with SCZ appear to give higher SoO^20,29^ and embodiment ratings^30,31^ than HC under asynchronous stimulation.

Regarding implicit illusion measures in the SCZ-S (e.g., proprioceptive drift), *k* = 9 studies applied implicit measures^13,20,29–32,46–48^, out of which *k* = 5 studies reported evidence for a greater proprioceptive drift in the synchronous conditions^13,29,31,46,48^, while *k* = 4 studies did not find an effect of synchronicity for their proprioceptive drift measure^30,32,47^ or global self-localization measure^20^.

### Research question 4: Are there associations between SCZ-S symptoms and BTI parameters?

*K* = 14 studies examined whether specific SCZ-S-related symptoms are correlated with BTI parameters. Out of those, *k* = 6 studies assessed subclinical symptoms in healthy individuals^13,31,38,46,47,49^ and *k* = 8 studies investigated symptoms in patients with SCZ or schizoaffective disorder^18,19,29,31,32,35,37,40^.

Regarding healthy participants, Thakkar et al.^31^ found moderate to large positive correlations of positive and negative schizotypy with SoO ratings in synchronous and asynchronous classical RHI conditions. Partly in line with this, Asai et al.’s^49^ RHI study found a moderate positive correlation between embodiment (composite score based on the proprioceptive drift and SoO ratings) and positive but not negative schizotypy. Similarly, psychoticism (interpreted as vulnerability for schizotypy) was found to correlate strongly with SoO in Kallai et al.’s^13^ classical RHI setup. Furthermore, Germine et al.^46^ detected a moderate positive correlation between positive psychosis-proneness and SoO under visuotactile synchrony, as well as between positive psychosis-proneness and SoA under both visuotactile synchrony and asynchrony. Similarly, applying a moving RHI, Louzolo et al.^38^ reported moderate positive correlations between delusion-proneness and SoO as well as between delusion-proneness and SoA during passive but not active movement. Finally, higher scores in psychotic-like experiences in healthy individuals screened for SCZ-S symptoms were found to be positively associated with an overall PHI rating (i.e., virtual limb embodiment and SoA; biological hand disembodiment and deafference)^47^. Notably, however, when analyzing all BTI components separately, this correlation only persisted for disembodiment of the biological hand.

Concerning SCZ patients, *k* = 3 studies reported moderate positive correlations between hallucination severity and different BTI parameters. Specifically, Peled et al.^35^ found a correlation with two SoO rating items, Rossetti et al.^19^ with SoO, SoA, and a hand location subscale, and Thakkar et al.^31^ for an overall SoO rating. Prikken et al.^29^, in turn, found a positive correlation between delusion severity and SoO during a synchronous RHI condition, while Ferri et al.^40^ found a positive correlation between anhedonia and SoO during a congruent condition in a modified RHI which focused on the anticipation of a touch experience. In contrast, *k* = 3 other studies^18,37,32^ did not find any significant correlations between BTI parameters and positive or negative symptom severity in patients with SCZ or schizoaffective disorder. Overall, the pattern of associations is therefore not clear, especially since the latter studies failed to detect significant associations between SCZ-S symptoms and BTI parameters.

## General discussion

This systematic review investigated whether SCZ-S populations show altered bodily self-awareness in BTI paradigms. More specifically, a systematic literature search in two databases was carried out, which identified *k* = 20 eligible studies. Overall, based on the four research questions addressed here, evidence points toward various alterations of bodily self-awareness in the SCZ-S: First, most studies reported that individuals within the SCZ-S tend to give higher SoO or embodiment ratings in visuotactile BTIs than HC^20,30–32,34–36^. Whether this effect also applies to visuomotor BTIs, however, remains unanswered, as no studies have been conducted on this issue so far. Second, individuals within the SCZ-S also appear to present alterations in their SoA. Unlike HC, who typically demonstrate reduced SoA after asynchronous compared to synchronous stimulation, individuals on the SCZ-S appear to report similar SoA levels (i.e., no statistical difference) under synchronous and asynchronous conditions^18,19,39^. Third, in line with results in HC, many studies found higher SoO^13,18–20,29,46,47^ and embodiment ratings^30–32^ after synchronous compared to asynchronous stimulation in individuals on the SCZ-S. However, some studies reported that individuals on the SCZ-S show higher SoO^20,29^ and embodiment ratings^30,31^ than HC during asynchronous conditions. Finally, the severity of positive SCZ symptoms (e.g., hallucinations), as well as higher scores of subclinical SCZ-S expressions (e.g., schizotypy) of the SCZ-S, appear to be associated with alterations of SoO and SoA^13,19,29,31,35,38,46,47,49^.

In the following, we first discuss the possibility raised by Shaqiri et al.^20^ that the observed results are merely methodological artifacts and do not represent specific changes in bodily self-awareness of SCZ patients. Next, assuming that the results are not merely methodological artifacts, we discuss these findings on a neurocognitive and phenomenological level, whereby both explanation attempts are not considered as competing, but as complementing each other at different levels of analysis.

### Interpretation of the results as a mere methodological artifact

Prior to the present systematic review, one meta-analysis had been carried out by Shaqiri et al.^20^ on SoO aberrations during BTI paradigms in individuals with SCZ. Interestingly, and at first glance in contradiction to the present results, Shaqiri et al. reported no evidence that SoO is elevated in individuals with SCZ. Of note, however, Shaqiri et al.^20^ applied rather strict criteria in their meta-analysis. Specifically, they exclusively focused on a two-way interaction between group (SCZ patients vs. HC) and condition (synchronous vs. asynchronous stimulation), based on the assumption that SoO aberrations in individuals with SCZ can only be demonstrated, if a group difference exclusively emerges under multisensory synchrony and not under multisensory asynchrony. Their underlying assumptions were (1) that multisensory asynchrony usually impairs SoO and (2) that increased SoO ratings could also be due to response bias unrelated to the actual experience. That is, individuals with SCZ, might show a generally increased confirmation bias and therefore report higher self-ratings on BTI paradigms than HC in general^20^. Given that Shaqiri et al.’s meta-study revealed no statistically significant interaction, they concluded that the hitherto reported “SoO differences” between SCZ patients and HC are more likely due to a general response bias of SCZ individuals rather than a specific SoO aberration.

While acknowledging this possibility, we consider it much more likely that at least part of the observed effects in fact reflects specific SoO aberrations in individuals with SCZ, for the following reasons. First, Shaqiri et al.’s meta-study was based on a small number of only four studies. Second, the question arises whether reporting SoO also during multisensory asynchrony actually constitutes sound evidence for a response bias. Just because healthy individuals usually report little SoO under multisensory asynchrony, it cannot be inferred that individuals from the SCZ-S have a similar experience. Instead, as discussed in the next section, individuals on the SCZ-S might for instance have an increased temporal binding window, that would explain the occurrence of SoO and SoA also after multisensory asynchrony.

In sum, our review cannot exclude the possibility that response biases had an impact on the observed results. On the other hand, we see no evidence that a response bias alone explains the results sufficiently, and assume that aberrations in SoO and SoA explain the data at least as well.

### Neurocognitive interpretation of the reported results

As reviewed and critically discussed by Klaver and Dijkerman^50^, various potential neurocognitive mechanisms have been proposed as potential explanations for higher SoO ratings of individuals on the SCZ-S during BTIs. Examples include an over-dominance of visual information, a longer temporal binding window, a stronger reliance on external sensory input than on pre-existing body representations, and a disturbed SoA, which in turn might affect SoO. As concluded by Klaver and Dijkerman^50^, the most convincing and unifying explanation is probably that patients with SCZ rely more heavily on multisensory information than on their stored body representations. According to this idea, the observed higher SoO ratings in SCZ-S individuals stem from the fact that SCZ-S individuals rely more heavily on their momentary visual input (and external stimuli in general) than on their proprioceptive input or pre-existing body representations. More specifically, during the BTI induction, SCZ-S individuals rely more on visual input (i.e., on the visual impression that the artificial hand is part of their own body) than on contradictory proprioceptive information concerning the position of their biological hand, or the consistency of their internal body representation (e.g., the deviating appearance of the artificial hand).

These explanations might also partially account for the finding of similar SoA under synchronous and asynchronous stimulation in SCZ-S individuals (see research question 2) and the occasionally persisting SoO under asynchronous stimulation (see research question 3). While Shaqiri et al.^20^ assumed that higher SoO scores after asynchronous stimulation in individuals with SCZ compared to HC are due to response biases, an alternative explanation would be longer temporal binding windows among SCZ-S individuals and a resultant reduced multisensory temporal acuity^50^. According to this hypothesis, the general and often replicated finding^51^ that individuals with SCZ show an increased time interval during which temporally separated sensory stimuli are still perceived as occurring simultaneously, also affects the BTI induction. More specifically, due to the enlarged temporal binding window, the multimodal asynchrony during asynchronous BTI conditions is possibly not (as much) encoded as asynchronous by SCZ individuals compared to HC^50^. Therefore, the influence of asynchrony on SoA and SoO is reduced in these subjects. In addition, regarding SoA, defective motor predictions have been associated with SoA persistence under multimodal asynchrony. According to this idea, SCZ is associated with impaired motor predictions that lead to difficulties in detecting kinematic dissimilarities caused by visuomotor incongruency; and therefore, SoA also persists under multimodal asynchrony^19^. Finally, the finding that a subclinical population high in delusion-proneness reported similar SoA ratings under active and passive movement has been attributed to a combination of reduced motor predictions and hypersalient processing of external input^38^. That is, for inferring agency, these individuals would rely less on their own motor predictions and intentions, but more on their sensory input perceived, and as a result experience SoA also during passive movement.

### Phenomenological interpretations

The reported findings can also be addressed from a phenomenological perspective. Referring to the aforementioned IDM, for instance, altered SoO demonstrated by SCZ-S populations during BTI exposure (cf. research question 1) might be a result of a disordered first-person perspective of experience (i.e., ipseity) characterized by three main aberrations: First, hyper-reflexivity. i.e., an excessive self-awareness in which aspects of oneself are experienced as alien and akin to external objects. Second, diminished self-presence, i.e., a reduced sense of being an agent of action and a reduced SoO toward everyday experiences^52^. And third, a disturbed “hold” or “grip” which refers to the loss of stability and salience of the field of awareness^4^. Based on these three aberrations of self-experience, higher SoO ratings of SCZ-S populations can be explained by a reduced self-demarcation. Accordingly, individuals on the SCZ-S might be more susceptible to perceive external objects (e.g., the artificial hand) as belonging to their own body due to diminished self-other boundaries.

Furthermore, the IDM also serves to explain the findings addressed in research question 2. Similar SoA ratings for synchronous and asynchronous stimulation in individuals on the SCZ-S hints toward an “over-inclusive” agency, as SoA ratings usually decrease in HC for asynchronous conditions. An “over-inclusive” agency could be the result of a reduced self-demarcation, making it more difficult for individuals on the SCZ-S to distinguish between their self and the external world. In addition, this could also be caused by a hyper-reflexivity (i.e., an exaggerated focus) on, in this case, external stimuli.

Finally, connecting the neurocognitive and phenomenological perspectives, Postmes et al.^53^ offered a parsimonious approach to explain the association between erroneous multisensory integration in BTIs and positive SCZ symptoms (cf. research question 4). According to the authors, conflicting sensory input (e.g., conflicting visual, tactile, and proprioceptive input as in the classic RHI paradigm) may lead to perceptual incoherence that could cause self-disorders as described in the IDM. For example, sensory imbalance or reduced sensory input promote hyperfocused attention. Simultaneously, it could be that unconscious efforts to regain perceptual coherence may result in delusions and hallucinations. Hence, the authors’ idea was that both increased BTI susceptibility and positive symptoms are secondary consequences of perceptual incoherence and therefore correlated.

### Limitations and future directions

One limitation is the rather low quality (RoB across studies: *M* = 60%; *SD* = 18.26%) of many conducted studies (for a detailed RoB listing, see Appendix 1). In fact, none of the *k* = 20 studies carried out a preregistration of their research project to reduce potentially problematic research practices (e.g., cherry-picking, p-hacking, or data dredging)^54^. Likewise, none of the studies applied a form of blinding (e.g., experimenter blinding) to avoid investigator effects. Also, *k* = 14 studies did not include control items into their questionnaire. The inclusion of such control items, however, appears crucial to control for possible response biases (e.g., extreme response bias, confirmation bias, social desirability bias) and to verify the illusion specificity of the experimental manipulations^21,55^ (for a critical discussion of control items, also see^56^). Similarly, *k* = 5 studies omitted the integration of an adequate control condition (e.g., an anatomically incongruent artificial hand condition or an asynchronous stimulation condition), which may help to rule out general responses biases^20^. Moreover, *k* = 6 studies did not perform adequate randomization or counterbalancing, implying that sequence effects cannot be excluded.

Another methodological weakness is that many studies^18,33–35,37–40^ did not include proprioceptive drift or other implicit measures to quantify the targeted artificial limb embodiment. However, as discussed above (cf. section “Theoretical background”), exploring implicit as well as explicit BTI measures appears particularly important, since both measures capture different aspects of artificial limb embodiment^44^. Moreover, the proprioceptive drift measure is probably less prone to general suggestibility effects and response biases than a BTI questionnaire. Therefore, future BTI research should include different implicit measures to operationalize the illusion. One promising approach is recording the participant’s electrodermal activity while the virtual hand is threatened by a virtual syringe, as implemented in some BTI studies^26,27,57^ and using this physiological marker as another implicit measure of artificial hand embodiment.

A further limitation concerns the interpretations of some of the studies, particularly regarding the questionnaire results. For example, among the studies that reported raw means of the questionnaire items, *k* = 6 studies^30,31,33,35,46,58^ found illusion scores ≤0 on a Likert scale ranging from −3 to +3 and still interpreted these scores as indicative of a successful BTI induction. Even if relative SoO differences might still be detectable despite weak embodiment (SoO) levels, the question arises, whether a meaningful manipulation of bodily self-awareness can still be assumed. A promising approach to counteract over-interpretations could be the integration of an absolute illusion threshold (e.g., +1 on a 7-point Likert scale, representing slight confirmation) in future research for a successful SoO and/or SoA induction.

Another limitation of the reviewed studies is that some of them reported strong interindividual differences within the SCZ-S, with illusion ratings ranging from strong disconfirmation to strong confirmation. Consequently, the samples acquired might not be sufficiently homogenous to justify a generalizable statement about bodily self-awareness within the whole spectrum of SCZ-related states. Instead, it appears that some BTI experiences are not explained by SCZ per se, but rather by subsets of SCZ-related symptoms. Three of the included studies in fact divided their SCZ samples into multiple subgroups according to symptomatology, such as current, past, and no experience of passivity symptoms^18,39^, or first-rank symptoms^37^. Focusing on specific aspects of the SCZ-S therefore appears to be a particularly interesting approach to study bodily self-awareness within the SCZ-S in further detail.

Moreover, concerning the fourth research question on associations between SCZ-S symptoms (e.g., schizotypy, psychosis-proneness, hallucinations) and BTI parameters, it has to be noted that none of the reviewed studies directly assessed self-disorders by means of dedicated assessment tools such as the interview-based Examination of Anomalous Self-Experiences (EASE)^59^ or the Inventory of Psychotic-Like Anomalous Self-Experiences (IPASE)^60^. To validate the hypothesis that SCZ is a disorder of the self and that BTI paradigms can contribute experimental evidence to this hypothesis, it is important to conduct thorough assessments of prevalent self-disorders in addition to the application of BTI paradigms.

Another aspect concerns the question of which conclusions can actually be drawn from the BTI results so far. Notably, most reviewed studies merely focused on the extent to which SCZ-S individuals are more prone to “experientially replace” a biological limb with an artificial one. That is, they all relied on BTI paradigms that expand already experienced SoO toward one’s biological limb to an artificial limb, but that do not instantiate any new limb in the participant’s body matrix or drastically change a limb’s perceptual appearance. Therefore, the question arises as to how much can actually be learned from observing BTI vulnerability differences and to what extent a higher (or lower) susceptibility to BTIs must be seen as pathological. Therefore, it might also be interesting to explore whether more drastic changes in bodily self-awareness can be induced and investigated in future studies. For instance, the effect of a “supernumerary limb illusion”, a modified RHI under which participants perceive more limbs than they physically possess^27,61^ could provide additional insight. If SCZ is indeed primarily caused by a “disunity of consciousness” (in the sense of an increased separation of individual ego parts), individuals with SCZ-S should be particularly vulnerable to this modified RHI.

Finally, an interesting application-oriented approach could be to investigate whether BTI paradigms prove useful as a therapeutic or psychoeducational element in patients with SCZ: Experiencing a BTI intuitively demonstrates that our bodily boundaries—which we usually take for granted and constant—can easily be modified within seconds. As a consequence, other SCZ-related symptoms such as shifts in the perception of reality (e.g., hallucinations, delusions) or reduced demarcation of inner and outer world (e.g., thought insertions), might be evaluated differently after experiencing a BTI in the therapeutic context.

The present systematic review has some limitations itself that must be taken into account. First, our literature search only included two databases, PubMed and CENTRAL, and only considered articles published in English. Therefore, it is possible that some publications were not identified. Second, a number of studies investigated SCZ and psychoses in healthy participants through the administration of substances such as ketamine^62,63^ or dexamphetamine^64^, which can induce psychosis-like experiences. The comparison of these studies with studies of patients on the SCZ-S remains difficult, which is why studies actively inducing psychosis-like symptoms were not included in the present review. Nevertheless, this approach represents a novel and promising way of conducting research in the SCZ-S. Third, to get an overall picture of the results, and because the number of published studies is still quite small, some interpretations refer to the whole SCZ-S and may ignore differences between populations. However, with more data available, a detailed comparison of populations within the SCZ-S with respect to BTI might be possible in the future. Finally, regarding the phenomenological underpinnings of possible alterations of bodily self-awareness presented here, we focus on the framework laid out by the IDM. It has to be noted that other phenomenological theories like the perceptual anomalies approach^65^ might offer additional insights into the subjective experience of BTIs. While also highlighting the potential over-salience of external stimuli, as well as describing the phenomenon of giving new meaning to what is experienced in SCZ individuals, this approach considers alterations in low-level, perceptual/automatic processing and resulting disruptions of the perceptual field at the core of these experiences^65^. As a consequence, it relies less on theoretical assumptions about the self (i.e., ipseity) and could be a more parsimonious framework to explain alterations of SoO and SoA.

## Conclusion

Our review indicates that during BTI exposure, populations on the SCZ-S demonstrate altered bodily self-awareness. Regarding SoO, especially SCZ patients appear to give stronger SoO ratings under most visuotactile BTI paradigms, whereas under visuomotor BTI paradigms results are less conclusive, presumably due to a lack of studies. Moreover, concerning implicit illusion measures, the majority of studies do not find higher SoO in SCZ-S populations. Regarding SoA, some studies report similar SoA levels after synchronous and asynchronous stimulation in SCZ individuals, while HC typically report significantly lower SoA under visuomotor asynchrony. Also, positive associations emerge between BTI measures and subclinical SCZ-S states (i.e., schizotypy, psychosis-, and delusion-proneness) as well as between BTI measures and SCZ symptoms (i.e., delusions and hallucinations). While on the neurocognitive level, a stronger influence of external stimuli or weaker stored body representations could be responsible for these findings, on the phenomenological level a reduced ego demarcation might be accountable. These findings, however, need to be interpreted with caution, given that many of the studies included in this review lacked sufficient methodological rigor. Therefore, further and more rigorous research is needed to identify possible pathomechanisms of bodily self-awareness in SCZ-S populations.

## Supplementary information

Risk of Bias Evaluation

## Data Availability

The whole literature search was conducted and documented in Mendeley. The relevant .bib-file documenting the conducted literature search will be made available by the authors, without undue reservation, to any qualified researcher.
