# Supplementary material for: Body transfer illusions in the schizophrenia spectrum: a systematic review
Source: Schizophrenia (Heidelb). 2022 Nov 23;8(1):103. doi: 10.1038/s41537-022-00314-z (PMC9691625; doi:10.1038/s41537-022-00314-z)
Supplement: Supplementary file 1 — Risk of Bias Evaluation [file 41537_2022_314_MOESM1_ESM.pdf]

Study	Study population		Paradigm	Study quality							
	N (male)	*Sample*		*RoB (%)*	*Randomization*	*Control-group*	*Control-condition*	*Control-items*	*Registration*	*Blinding*	*Overall*
Peled et al. (2000)	SCZ = 26 (20)<br>HC = 23 (10)	Adults with schizophrenia	Classical RHI	83	-	+	-	-	-	-	1
Peled et al. (2003)	SCZ = 19 (16)<br>HC = 19 (17)	Adults with schizophrenia	Classical RHI	83	-	+	-	-	-	-	1
Asai et al. (2011)	72 (36)	Healthy adults	Classical RHI	83	+	-	-	-	-	-	1
Thakkar et al. (2011)	SCZ = 24 (15)<br>HC = 21 (11)	Adults with schizophrenia	Classical RHI	50	+	+	+	-	-	-	3
Germine et al. (2013)	55 (20)	Healthy adults	Classical RHI	83	-	-	+	-	-	-	1
Ferri et al. (2014)	SCZ = 21 (21)<br>HC = 17 (17)	Adults with schizophrenia	RHI derivate (expectation of touch)	50	+	+	+	-	-	-	3
Graham, Martin-Iverson, Holmes, Jablensky, et al. (2014)	SCZ = 53 (36); HC = 48 (24)	Adults with schizophrenia or schizoaffective disorder – sub-division: never, past or current passivity symptoms	Projected Hand Illusion	50	+	+	+	-	-	-	3
Graham, Martin-Iverson, Holmes, & Waters (2014)	HC = 48 (24)	Healthy adults	Projected Hand Illusion	67	+	-	+	-	-	-	2
Kaplan et al. (2014)	SCZ = 17 (4)<br>BDD = 17 (4)<br>HC = 17 (4)	Adults with schizophrenia/ schizoaffective disorder and adults with BDD	Classical RHI	33	+	+	+	+	-	-	4
Kállai et al. (2015)	HC = 48 (20)	Healthy adults	Classical RHI	33	+	-	+	+	-	+	4

Lev-Ari et al. (2015)	SCZ = 30 (24); HC = 30 (15)	Adults with schizophrenia	Classical RHI	83	-	+	-	-	-	-	1
Louzolo et al. (2015)	HC = 71 (30)	Healthy adults	Moving RHI	50	+	-	+	+	-	-	3
Mirucka (2016)	SCZ = 31(17); HC = 33 (10)	Adults with schizophrenia	Classical RHI	83	-	+	-	-	-	-	1
Graham-Schmidt et al. (2018)	SCZ = 51(35); HC = 47(24)	Adults with schizophrenia or schizoaffective disorder - sub-division: never, past or current passivity symptoms	Projected Hand Illusion	67	-	+	+	-	-	-	2
Shaqiri et al. (2018)	SCZ = 59 (44); HC = 30 (16)	Adults with schizophrenia	Full Body Illusion	33	+	+	+	+	-	-	4
Prikken et al. (2019)	Cohort 1: SCZ = 54 (46); HC = 56 (52)<br>Cohort 2: SCZ-risk = 24 (6); Mood disorder risk = 33 (15); HC children = 18 (13)	Cohort 1: Adults with schizophrenia<br>Cohort 2: Children / adolescents with increased familiar risk of schizophrenia and children / adolescents with increased familiar risk of mood disorder	Classical RHI	50	+	+	+	-	-	-	3
Costantini et al. (2020)	SCZ = 22 (22); HC = 22 (22)	Adults with schizophrenia	Classical RHI	67	+	-	+	-	-	-	2
Rossetti et al. (2020)	SCZ = 29 (18); HC = 36 (6)	Adults with schizophrenia	Mirror Box	50	+	+	+	-	-	-	3
Zopf et al.,(2020)	SCZ = 23 (15); HC = 21 (12)	Adults with schizophrenia or schizoaffective disorder	Classical RHI + LED/tactor stimulation	50	+	+	+	-	-	-	3
Laurin et al. (2021)	FRS+ = 31 (23)<br>FRS- = 25 (11)	Adults with schizophrenia with first rank symptoms (FRS+) and without first rank symptoms (FRS-)	Moving RHI	50	+	-	+	+	-	-	3
